# Short-term effect of ovariohysterectomy on urine serotonin, cortisol, testosterone and progesterone in bitches

**DOI:** 10.1186/s13104-021-05680-y

**Published:** 2021-07-10

**Authors:** Eva Hydbring-Sandberg, Elin Larsson, Andrzej Madej, Odd Viking Höglund

**Affiliations:** 1 Department of Anatomy, Physiology and Biochemistry, Swedish University of Agricultural Sciences, PO Box 7011, 750 07 Uppsala, Sweden; 2 Department of Clinical Sciences, Swedish University of Agricultural Sciences, PO Box 7054, 750 07 Uppsala, Sweden

**Keywords:** Bitches, Canine, Cortisol, Gonadectomy, Neuter, Progesterone, Serotonin, Spay, Testosterone, Urinary hormones

## Abstract

**Objective:**

This study aimed to investigate the short-term effect of ovariohysterectomy on urine levels of serotonin and its relation to levels of cortisol, testosterone and progesterone in female dogs. Seven bitches were studied before surgical ovariohysterectomy and then once a week during 4 weeks. Spontaneously voided urine samples were collected and concentration ratios of hormone/creatinine in urine were analysed.

**Results:**

The bitches had significantly lower levels of cortisol, testosterone, and progesterone 1 week after ovariohysterectomy compared with before and the levels stayed low throughout the study (P ≤ 0.05). Interestingly, serotonin levels tended to increase 4 weeks after surgery (P = 0.08). A positive correlation between cortisol and progesterone was found before and after surgery. After surgery, serotonin was positively correlated with cortisol and progesterone (P ≤ 0.05).

**Supplementary Information:**

The online version contains supplementary material available at 10.1186/s13104-021-05680-y.

## Introduction

Dog owners may have their dogs neutered to reduce specific behaviours or to resolve persisting behaviour problems [1]. Higher cortisol levels have been associated with different types of stress situations [2] as well as aggression [3] and fear [4, 5]. Lower cortisol levels in gonadectomized dogs compared to intact dogs have been found in saliva [6], as well as in serum [7]. However, other studies found no such differences in circulating cortisol concentrations [8, 9]. Serotonin levels in dogs have been inversely correlated with aggression [3, 10–12] and impulsivity [13]. Expression of serotonin transporter (5-HTT) in peripheral blood mononuclear cells was positively correlated with plasma cortisol in infant rhesus macaques [14]. Lower levels of plasma/serum testosterone have been reported in castrated male dogs compared to intact dogs [8, 15, 16]. Aggressiveness was found to be related to testosterone level in male dogs [17], male Ethiopian wolves [18] and boars [19]. GnRH agonist slow-release implants, that inhibit testosterone production, play an important role in the reduction of aggressiveness in male dogs [17] and even might have impact on behaviour of female dogs [20]. It is known that in bitches, the ovaries are sites of testosterone production [14, 21]. Recently, in a study on women, the authors suggested that an aggressive response to provocation was related to high levels of testosterone in saliva [22]. Plasma progesterone levels have been reported to be lower in neutered than intact dogs of both genders [8]. To our knowledge there is only one publication concerning changes of urinary concentration of serotonin in gonadectomized dogs, without information about the time between surgery and experiment [23]. In rats an increased function of hypothalamic serotonin-1A receptor was seen after ovariectomy [24]. Thus, there is no previous data about the effect of gonadectomy on serotonin levels in dogs. The objective of this study was therefore to investigate the effect of short-term ovariohysterectomy on urine levels of serotonin and its relation to levels of cortisol, testosterone and progesterone in female dogs. The hypothesis was that gonadectomy affects the serotonin concentrations and if a possible correlation with other hormones exists.

## Main text

### Methods

#### Animals

Seven privately owned bitches (mean 3.6 years) of different breeds were recruited at three animal veterinary clinics (see Additional file 1: Table S1). The study only included dogs whose owners had already decided to have their dogs neutered before accepting to participate in this study.

The bitches were in late diestrus or early anestrus (mean 4.1 months after heat) and none had previously been chemically neutered nor given pharmaceuticals to delay or disrupt heat (see Additional file 1: Table S1). Surgical procedures were done at three clinics in Uppsala, Sweden. All bitches were subjected to ovariohysterectomy through ventral midline incision. No dog had fever or depressed general condition or was on regular medication before or after gonadectomy except for postoperative analgesic prescription from their respective veterinarian. Moreover, one dog was treated with gastro-protective sucralfate for 7 days after surgery (see Additional file 1: Table S1). Participation in this study did not affect the ordinary treatment of the dogs before, during, and after surgery except for urine sampling. Urine sampling was approved by the Animal Ethics Committee in Uppsala (C287/12); the use of privately owned animals by the Swedish Board of Agriculture (Dnr 31-11700/12).

#### Urine samples

Spontaneously voided urine samples were collected on six occasions by the owner: the evening before surgery, the morning of surgery, and every seventh morning for the next 4 weeks. Sample 1 was collected during the previous evening dog walk and all other samples were collected during the first morning dog walk, prior to the dog’s first meal. The urine was transferred into dark coloured tubes containing 3.2 M HCl (150 µL/mL), to improve sustainability by acidification to optimize the serotonin analysis. Urine was also transferred to empty test tubes for analysis of creatinine, cortisol, testosterone and progesterone. Samples were stored at − 20 °C during the experiment, and at − 70 °C when the last sample was collected until analysis.

#### Analyses

Creatinine was quantified by Arbor Assays Urinary Creatinine Detection Kit (Michigan, USA). MDV was determined to 15.1 μmol/L and the intraassay CV was < 10% between 53 and 1768 μmol/L. The analyses of each respective hormone were performed on 2 different days, with samples from one half of the group analysed on each day. All hormones were analysed by ELISA according to manual guidelines. Cortisol was quantified by IBL International Urine Cortisol ELISA (Hamburg, Germany), serotonin by IBL International Serotonin ELISA (Hamburg, Germany), testosterone by Testosterone Arbor Assays Nordic Biosite, and progesterone by Progesterone Arbor Assay Nordic Biosite (Stockholm, Sweden). Due to one missing sample, six samples were analysed for serotonin from the morning before surgery (sample 2). Analytical data are presented in Additional file 2: Table S2.

#### Statistics

Physiological data were analysed with SAS Software 2008 (Statistical Analysis Systems, 9.2, SAS Institute, Cary, NC, USA) and presented as mean values for each sample (SEM) ± Standard Errors (SE). ANOVA (Mixed Procedure) were used to study whether samples from bitches differed significantly. Samples taken the morning before surgery were chosen as controls, and designated as the reference to which all samples collected post-surgery were compared. The statistical model included the fix effect of sample and the random effect of individual. The Pearson correlation coefficient was applied to investigate correlations between the hormones before (sample 1–2) and after (sample 3–6) ovariohysterectomy. The significance level was set at P ≤ 0.05.

### Results

#### Serotonin

**Fig. 1:**
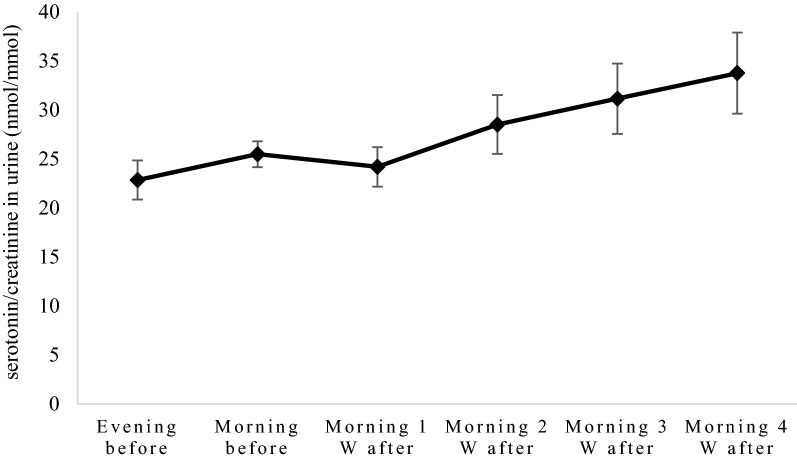
Serotonin/creatinine ratios (mean value ± standard error) in seven bitches before and after ovariohysterectomy. W = weeks. The morning sample before surgery included only six bitches due to problems with collecting. The serotonin levels did not differ significantly between the morning before ovariohysterectomy and the other samples. Four weeks after surgery the levels tended to be higher than that before (P = 0.08)

Before and 1 week after ovariohysterectomy, the concentration ratio of serotonin/creatinine in urine varied between 22.8 and 24.2 nmol/mmol. Afterward, an increase of concentration ratio of serotonin/creatinine was observed. Four weeks after surgery the concentration ratio was 33.7 nmol/mmol, which tended to be higher (P = 0.08) than the morning before (Fig. 1).

#### Cortisol

**Fig. 2:**
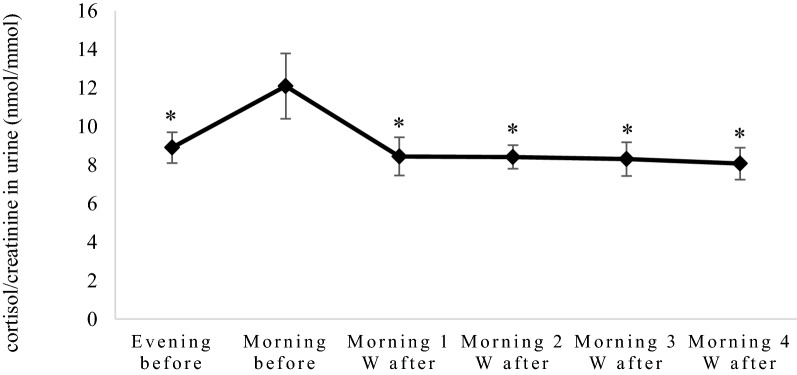
Cortisol/creatinine ratios (mean value ± standard error) in seven bitches before and after ovariohysterectomy. W = weeks. The cortisol level was higher the morning before ovariohysterectomy compared with all samples after ovariohysterectomy and the evening before. *Significantly different from morning sample before ovariohysterectomy. P ≤ 0.05

The urinary cortisol/creatinine ratio in the morning sample was significantly higher than in the evening sample before surgery (12.1 vs. 8.9 nmol/mmol, respectively, P ≤ 0.05). Ovariohysterectomy resulted in a significant decrease of urinary cortisol in the morning samples within 1 week after surgery (from 12.1 to 8.44 nmol/mmol, P ≤ 0.05). The low concentrations of cortisol were observed during the entire experimental period and were not different from the concentration in the evening sample before ovariohysterectomy (Fig. 2).

#### Testosterone and progesterone

**Fig. 3:**
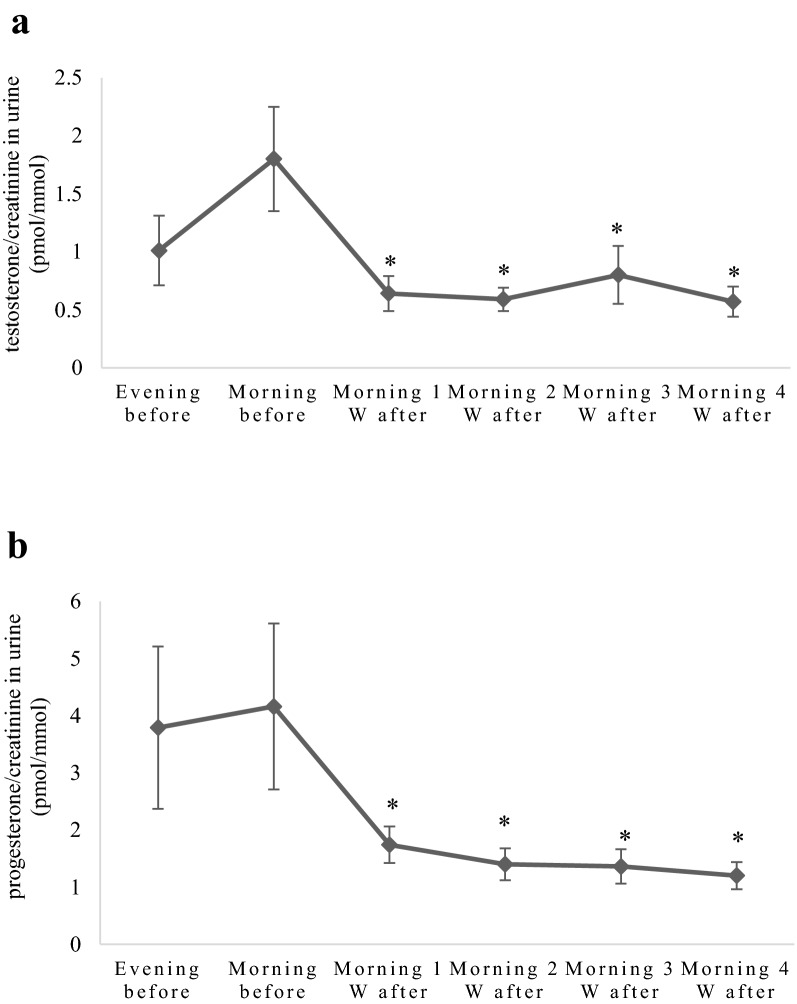
**a** Testosterone/creatinine ratios (mean value ± standard error) in seven bitches before and after ovariohysterectomy. W = weeks. The testosterone levels decreased after ovariohysterectomy and stayed low in all samples. Evening and morning ratios before ovariohysterectomy did not differ. *Significantly different from morning sample before ovariohysterectomy. P ≤ 0.05. **b** Progesterone/creatinine ratios (mean value ± standard error) in seven bitches before and after ovariohysterectomy. W = weeks. The progesterone level decreased after ovariohysterectomy and stayed low in all samples. Evening and morning ratios before ovariohysterectomy did not differ. *Significantly different from morning sample before ovariohysterectomy. P ≤ 0.05

Before ovariohysterectomy, the urinary testosterone/creatinine ratio was 1.8 pmol/mmol. After surgery, a dramatic significant decrease in testosterone concentration was seen achieving 0.57 pmol/mmol 4 weeks later. Evening and morning samples did not differ before surgery (Fig. 3a). The urinary progesterone/creatinine ratio significantly decreased after surgery from 4.16 pmol/mmol and stayed low between 1.2 and 1.74 pmol/mmol throughout the experimental period. Evening and morning samples did not differ. Individual levels diverged more before than after surgery (Fig. 3b).

#### Correlations between hormones

There was a positive correlation between cortisol and progesterone before and after surgery (r = 0.72, P = 0.004 and r = 0.67, P = 0.0001, respectively). After surgery serotonin was positively correlated with cortisol (r = 0.44, P = 0.02) and progesterone (r = 0.38, P = 0.05). See Additional file 3: Table S3.

### Discussion

To our knowledge, this is the first study about the short-term effect of ovariohysterectomy on serotonin levels in bitches. In the present study, the urinary serotonin/creatinine ratio in bitches tended to be higher 4 weeks after surgery. In addition, serotonin was positively correlated with both cortisol and progesterone but not with testosterone after ovariohysterectomy. In male rats, castration increased serotonin-1A messenger RNA content in the cortex, hypothalamus, hippocampus, and amygdala [25], which may indicate an inverse relationship between testosterone and serotonin. A positive correlation between post-stress cortisol measures and serotonin 1A receptor ligand binding levels across multiple cortical and subcortical regions was reported in humans [26]. It was suggested that both serotonin and cortisol are associated in sex-differentiated responses to stress and possibly to anxiety, as they both rise in response to stress, especially in women [27]. The positive correlation between serotonin and cortisol found in our study was in agreement with previous report that serotonin affect the secretion of CRH and ACTH at the hypothalamic, pituitary gland level, and possibly also at the adrenal gland level [28]. In addition, serotonin, microinjected to paraventricular nucleus of conscious rats, activated the CRH synthesis, which resulted in increased plasma ACTH [29]. A plausible explanation for the positive correlation between serotonin and progesterone is that progesterone is secreted from adrenal glands after ovariohysterectomy. The effect of ACTH stimulation in ovarioectomized bitches have earlier been reported [8]. Recently, interbreed variation of serum serotonin in healthy dogs was reported [30]. The authors also found that males had higher levels of serotonin in serum than bitches [30]. Urine serotonin/creatinine ratio in our ovariohysterectomized bitches varied approximately between 20 and 40 nmol/mmol and was somewhat lower than in gonadectomized dogs of both genders (i.e. between 50 and 90 nmol/mmol) [23]. It is difficult to explain the reason for the higher serotonin levels in ovariectomized bitches. The present data may be important for power analysis in future studies and therefore serves as important reference material. Further studies are required to evaluate why and what effect this may have on the individual dog.

The bitches had lower cortisol levels 1 week after surgery than the morning before, and these levels remained low. Reportedly, the circadian rhythm of cortisol secretion results in high concentrations in the morning and low in the evening [31, 32]. Furthermore, the circadian rhythm in plasma cortisol has been reported to be disrupted in old dogs and not yet developed in puppies [31]. However, other authors found no circadian rhythm in cortisol in male dogs [33, 34] and bitches [34]. In agreement with our findings, saliva cortisol levels have been found to be lower in spayed bitches than in intact bitches [6] and lower serum cortisol levels have also been found in bitches 7–15 days after spaying than before [7]. Other studies found that plasma cortisol levels did not differ but they did not study the same individuals before and after gonadectomy [8, 9].

In our study, we found that ovariohysterectomy in bitches dramatically decreased urinary testosterone. This is in contrast to a study where plasma testosterone did not differ between intact and spayed females [8]. However, our results agreed with other studies that found basal plasma testosterone levels significantly higher before than after gonadectomy in female dogs [15]. These authors also reported that GnRH administration before gonadectomy caused significantly increased plasma testosterone levels in bitches. This observation agrees with another report that suggested that the ovaries may be sites of testosterone production in female dogs [21]. Thus, our findings provide further evidence that testosterone originates mainly from the ovaries in bitches.

In the present study, the progesterone levels decreased significantly 1 week after gonadectomy and stayed low throughout the experiment. Plasma progesterone levels have been reported to be lower in neutered dogs of both genders [8].

The relation between hormones and behaviour is complex and was outside stipulated aims of the study. However, measurements of serotonin, cortisol, testosterone and progesterone in urine collected by a non-invasive process may be useful for understanding of both female and male dogs’ behaviour after gonadectomy.

## Limitations

The number of dogs was relatively small and the bitches were of different breeds and in different phases of estrous cycle.

## Supplementary Information

**Additional file 1: Table S1.** Participating dogs.

**Additional file 2: Table S2.** Hormonal analyses.

**Additional file 3: Table S3.** Hormonal correlations.

## Data Availability

The datasets used and/or analysed during the current study are available from the corresponding author on reasonable request.
