# Supplementary material for: Short-term effect of ovariohysterectomy on urine serotonin, cortisol, testosterone and progesterone in bitches
Source: BMC Res Notes. 2021 Jul 10;14:265. doi: 10.1186/s13104-021-05680-y (PMC8272283; doi:10.1186/s13104-021-05680-y)
Supplement: Supplementary file 1 — Additional file 1: Table S1. Participating dogs. [file 13104_2021_5680_MOESM1_ESM.docx]

**Table S1. Description of the participating dogs**

|  | **Bitch and breed** | **Age (years)** | **Time from heat (months) and**  **reason for gonadectomy** | **Postoperative analgesics and time in home environment** |
| --- | --- | --- | --- | --- |
|  | Bitch A  *Golden retriever* | 7 | 1,5  *Medical reasons, prolonged heat and abnormal findings on uterus at ultrasound examination* | Carprofen, 3 days |
|  |  |  |  | |
|  | Bitch B  *Coton de Tuléar* | 2 | 8  *Problematic dieostrus periods* | Buprenorphine, 2 days |
|  |  |  |  |  |
|  | Bitch C  *Jack Russel Terrier* | 6 | 6  *Problematic dieostrus periods* | Meloxicam, 5 days |
|  |  |  |  |  |
|  | Bitch D  *Chinese Crested Dog* | 4 | 5  *Problematic heat periods* | Meloxicam, 7 days |
|  |  |  |  |  |
|  | Bitch E  *Swedish vallhund* | 1 | 4  *Medical reasons; dermatological issues and vaginal discharge* | Meloxicam, 4 days |
|  |  |  |  | |
|  | Bitch F  *Chihuahua, long-haired* | 1 | 2,5  *Prevent reproduction* | Meloxicam, 7 days |
|  |  |  |  |  |
|  | Bitch G  *Mixed breed* | 4 | 2  *Problematic dieostrus periods* | Carprofen, 4 days |
|  |  |  |  |  |
| Average |  | 3.6 years | 4.1 month (1.5-8 months) | 4.6 days |
