# Supplementary material for: Short-term effect of ovariohysterectomy on urine serotonin, cortisol, testosterone and progesterone in bitches
Source: BMC Res Notes. 2021 Jul 10;14:265. doi: 10.1186/s13104-021-05680-y (PMC8272283; doi:10.1186/s13104-021-05680-y)
Supplement: Supplementary file 2 — Additional file 2: Table S2. Hormonal analyses. [file 13104_2021_5680_MOESM2_ESM.docx]

## **Table S2. Analytical data of hormonal analysis**

|  | **Minimum detectable value (MDV)** | **Intra-assay CV** | **Recovery**  **(%)** | **Inter-assay CV**  **(2 assays)** |
| --- | --- | --- | --- | --- |
| Serotonin | 80.1 nmol/L | <10 % (482-12016 nmol/L) | 87 | 0 % at 618 nmol/L,  0.6 % at 1511 nmol/L |
| Cortisol | 7.3 nmol/L | <10% (27-1380 nmol/L) | 112 | 0 % at 110 nmol/L,  2.2 % at 688 nmol/L |
| Testosterone | 51.7 pmol/L | <10 % (479- 34700 pmol/L) | 86 | 19.9 % at 8466 pmol/L,  10.3 % at 17127 pmol/L |
| Progesterone | 151.3 pmol/L | <10% (292- 10176 pmol/L) | 114 | 9.3 % at 868 pmol/L,  0.4 % at 3688 pmol/L |
