# Supplementary material for: Short-term effect of ovariohysterectomy on urine serotonin, cortisol, testosterone and progesterone in bitches
Source: BMC Res Notes. 2021 Jul 10;14:265. doi: 10.1186/s13104-021-05680-y (PMC8272283; doi:10.1186/s13104-021-05680-y)
Supplement: Supplementary file 3 — Additional file 3: Table S3. Hormonal correlations. [file 13104_2021_5680_MOESM3_ESM.docx]

**Table S3. Hormonal correlations* before (sample 1-2) and after (sample 3-6) ovariohysterectomy**

|  | Serotonin | Cortisol | Testosterone | Progesterone |
| --- | --- | --- | --- | --- |
| Serotonin  Before  After |  | r=0.26  P=0.39  **r=0.44**  **P=0.02** | r=-0.08  P=0.79  r=0.21  P=0.28 | r=0.33  P=0.27  **r=0.38**  **P=0.05** |
| Cortisol  Before  After | r=0.26  P=0.39  **r=0.44**  **P=0.02** |  | r=0.46  P=0.10  r=-0.04  P=0.84 | **r=0.72**  **P=0.004**  **r=0.67**  **P=0.0001** |
| Testosterone  Before  After | r=-0.08  P=0.79  r=0.21  P=0.28 | r=0.46  P=0.10  r=-0.04  P=0.84 |  | r=0.47  P=0.09  r=0.24  P=0.21 |
| Progesterone  Before  After | r=0.33  P=0.27  **r=0.38**  **P=0.05** | **r=0.72**  **P=0.004**  **r=0.67**  **P=0.0001** | r=0.47  P=0.09  r=0.24  P=0.21 |  |

*Pearson Correlation Coefficients. Bold figures indicate significant differences. P ≤ .05.
